# Liver injury with COVID-19 based on gastrointestinal symptoms and pneumonia severity

**DOI:** 10.1371/journal.pone.0241663

**Published:** 2020-11-04

**Authors:** Shun Kaneko, Masayuki Kurosaki, Kaoru Nagata, Reiko Taki, Ken Ueda, Satoko Hanada, Koji Takayama, Shinichiro Suzaki, Naoshige Harada, Toru Sugiyama, Masayuki Nagasawa, Namiki Izumi

**Affiliations:** 1 Department of Gastroenterology and Hepatology, Musashino Red Cross Hospital, Tokyo, Japan; 2 Department of General Medicine, Musashino Red Cross Hospital, Tokyo, Japan; 3 Department of Pulmonary Medicine, Musashino Red Cross Hospital, Tokyo, Japan; 4 Department of Emergency and Intensive Care, Musashino Red Cross Hospital, Tokyo, Japan; 5 Department of Endocrinology and Metabolism, Musashino Red Cross Hospital, Tokyo, Japan; 6 Division of Infection Control and Prevention, Musashino Red Cross Hospital, Tokyo, Japan; Nihon University School of Medicine, JAPAN

## Abstract

**Background/Aim:**

The coronavirus disease 2019 (COVID-19) had become a big threat worldwide. Liver injury is not uncommon in patients with COVID-19, and clarifying its characteristics is needed. This study aimed to identify factors associated with liver injury and to develop a new classification of predictive severity in patients with COVID-19.

**Methods:**

Confirmed patients with COVID-19 (n = 60) were recruited retrospectively from Musashino Red Cross Hospital. The factors of liver injury especially on the elevation of liver enzymes (aspartate aminotransferase [AST] and alanine aminotransferase [ALT]) were analyzed. Grading was assessed according to the Common Terminology Criteria for Adverse Events (CTCAE) version 5.0.

**Results:**

During a median hospitalization follow-up of 15 (4–41) days, 51 (85.0%) patients had COVID-19 pneumonia. In clinical courses, oxygenation was needed for 25 (41.6%) patients and intubation was needed for 9 (15.0%) patients. A total of 27 (45.0%) patients had gastrointestinal symptoms (GS), such as appetite loss, diarrhea, and nausea. A logistic regression analysis revealed that C-reactive protein (CRP) at baseline, oxygenation, intubation, and GS were significant factors of liver injury. Based on these results, patients were classified into three groups: group 1, no oxygenation pneumonia; group 2, pneumonia with oxygenation or GS; and group 3, intubation. We classified 25 (41.7%), 26 (43.3%), and 9 (15.0%) patients into mild, moderate, and severe groups, respectively. The peak of AST and ALT levels was significantly stratified with this criteria (mild [median AST, 28 IU/L; median ALT, 33 IU/L], moderate [median AST, 48 IU/L; median ALT, 47.5 IU/L], and severe [median AST, 109 IU/L; median ALT, 106 IU/L]; *P*<0.001 and *P* = 0.0114, respectively).

**Conclusion:**

COVID-19-related liver injury was significantly stratified based on GS and severity of pneumonia.

## Introduction

The coronavirus disease 2019 (COVID-19) was first reported in Wuhan, China, in December 2019, and the COVID-19 outbreak had become a big threat worldwide. As of June 19, 2020, more than 8.38 million cases of COVID-19 and more than 450,000 deaths had been reported worldwide [1]. Severe acute respiratory syndrome coronavirus 2 (SARS-CoV-2), a new strain of coronavirus that has not been previously identified in humans, is the cause of COVID-19 [2]. The common symptoms of COVID-19 include respiratory symptoms, fever, and breathing difficulties [3]. In more severe cases, it causes pneumonia, acute respiratory distress syndrome, kidney failure, acute cardiac injury, multi-organ failure, and even death [4–7].

Gastrointestinal symptoms (GS) and liver injury in patients with COVID-19 have been increasingly reported [3]. In a meta-analysis study, GS and liver injury are not uncommon in patients with COVID-19 [8]. Some reports suggested that patients with severe disease tend to have an increased risk of developing GS and abnormal liver function [9–12]. However, the results have some inconsistencies with heterogeneity. Because COVID-19 is a disease in which pneumonia is a main focus, studies about GS and liver injury were mainly based on subgroup analysis. Since detailed observational studies are not yet enough, to clarify the actual condition of liver injury especially in terms of liver enzyme elevation in patients with COVID-19, we conducted this detailed observational study.

This study aimed to reveal factors associated with liver injury and to develop a new classification for predictive liver injury. Moreover, we assessed the relationship between GS and not only liver enzyme elevation but also biliary enzyme and inflammatory markers in patients with COVID-19.

## Method

### 1. Patients

Confirmed patients with COVID-19 were recruited retrospectively from Musashino Red Cross Hospital between March and May 2020. All patients were examined by reverse transcription polymerase chain reaction (RT-PCR) for SARS-CoV-2 using pharyngeal and nasopharyngeal swabs collected in accordance with the nationally recommended method in Japan [13]. This study was approved by the Ethical Committee of Musashino Red Cross Hospital that was conducted in accordance with the Declaration of Helsinki (confirmation number: 2020–448).

### 2. Clinical evaluation

Clinical information including previous history was collected from the medical records. The age and gender of patients were recorded at the time of study entry. Hepatitis B virus, hepatitis C virus, and human immunodeficiency virus tests were performed at admission. As the definition of the days from onset, most of the patients had clinical symptoms such as fever, cough, diarrhea, malaise, and tachypnea. We defined the days of serum sample collection after the onset of the first symptoms. In the asymptomatic patients, the first day of positive RT-PCR result for upper respiratory specimens was determined as the day of onset. The diagnosis of COVID-19 pneumonia was based on a chest computed tomography showing a typical ground-glass opacity, consistent with a previous report [3, 14]. The grade of liver enzyme elevation was assessed using the Common Terminology Criteria for Adverse Events (CTCAE) version 5.0 [15] as follows: grade 1 (male: aspartate aminotransferase [AST], 41–120 U/L; alanine aminotransferase [ALT], 34–99 U/L; female: AST, 33–96 U/L; ALT, 34–99 U/L), grade 2 (male: AST, 121–200 U/L; ALT, 166–275 U/L; female: AST, 97–160 U/L; ALT, 100–165 U/L), and grade 3 (male: AST, >201 U/L; ALT, >276 U/L; female: AST, >161 U/L; ALT, >166 U/L).

### 3. Statistical analyses

Chi-square and Fisher’s exact tests were used to compare categorical variables. Student’s *t*-test, Mann-Whitney *U* test, or Kruskal-Wallis test was used to analyze the distribution of continuous variables. Logistic regression analysis was used to analyze factors associated with liver enzyme elevation. Significance was set at *P*<0.05. The GraphPad Prism software (GraphPad Software, San Diego, CA, USA) and EZR (Saitama Medical Center, Jichi Medical University, Shimotsuke, Japan) were used to analyze statistical significance.

## Results

### 1. Patient characteristics

A total of 60 patients with COVID-19 who were admitted at Musashino Red Cross Hospital between January and May 2020 were included in this study. All patients were confirmed of COVID-19 by RT-PCR for SARS-CoV-2 using pharyngeal and nasopharyngeal swabs. Among them, 39 (65.0%) patients were male; the mean age was 54.1 ± 14.7 years; 10 (16.6%) patients were diagnosed with diabetes mellitus; no patients had chronic viral hepatitis (hepatitis B and C virus) and cirrhosis. The baseline serum data (platelet, albumin, bilirubin, AST, ALT, alkaline phosphatase, creatinine, and C-reactive protein [CRP]) are presented in Table 1. The median follow-up duration was 15 (4–41) days as hospital stay.

**Table 1:** Characteristics of patients admitted with COVID-19.

	n=60
Male gender (n,%)	39 (65.0%)
Age (years)	54.1±14.7
Diabetes mellitus (n,%)	10 (16.6%)
Chronic viral hepatitis (n) HBV/HCV/Other	0/0/0
Cirrhosis (n,%)	0 (0%)
Platelet (x10^9^/L)	220±73.9
Albumin (g/dl)	3.7±0.6
Total bilirubin (mg/dl)	0.57±0.30
Aspartate transaminase (IU/L)	53.2±62.6
Alanine aminotransferase (IU/L)	47.4±52.7
Alkaline phosphatase (IU/L)	253.7±158.1
Creatinine (mg/dl)	0.88±0.69
C reactive protein (mg/dl)	6.09±7.96
Pneumonia (n,%)	51 (85.0%)
Oxygenation (n,%)	25 (41.6%)
Intubation (n,%)	9 (15.0%)
Gastrointestinal symptoms (n,%)	27 (45.0%)
Appetite loss (n,%)	22 (36.7%)
Diarrhea (n,%)	12 (20.0%)
Nausea (n,%)	3 (5.0%)
Antibiotic (n,%)	41 (68.3%)
Investigational agent (n,%)	
Ciclesonide	32 (53.3%)
Favipiravir	20 (33.3%)
Hydroxychloroquine	5 (8.3%)
Hospital stay duration (days)	15(4-41)

Date are presented as mean±SD.

Abbreviations: HBV, hepatitis B virus; HCV, hepatitis C virus.

A total of 51 (85.0%) patients had COVID-19 pneumonia. In clinical courses, oxygenation was needed for 25 (41.6%) patients and 9 (15.0%) patients required intubation and ventilator management. During the follow-up, 2 (3.3%) patients died.

As investigational agents, 32 (53.3%), 20 (33.3%), and 5 (8.3%) patients received ciclesonide, favipiravir, and hydroxychloroquine, respectively.

### 2. Factors associated with liver injury

In addition to the baseline characteristics used for analyzing factors associated with liver injury, we also focused on GS; 27 (45.0%) patients had GS, such as appetite loss (22 [36.7%]), diarrhea (12 [20.0%]), and nausea (3 [5.0%]).

Logistic regression univariate analysis was used to investigate the predictive factors of liver injury and its degree. In patients with CTCAE grade of >1, older age, creatinine, CRP at baseline, oxygenation, intubation, and GS were significant factors of liver injury. In patients with CTCAE grade of >2, CRP at baseline (odds ratio [OR], 1.170; 95% confidence interval [CI], 1.06–1.30; *P* = 0.003), intubation (OR, 11.5; 95% CI, 2.31–57.3; *P* = 0.003), and GS (OR, 6.530; 95% CI, 1.25–34.0; *P* = 0.026) were independent factors of liver injury (Table 2). Oxygenation was marginally a significant factor (OR, 4.150; 95% CI, 0.953–18.1; *P* = 0.058).

**Table 2:** Logistic regression analysis for factors associated with liver injury in COVID-19 patients.

	>Grade 1			>Grade 2		
	OR	95%CI	p value	OR	95%CI	p value
Age (years)	1.060	1.02-1.110	0.007	1.000	0.957-1.05	0.918
Male gender	1.200	0.408-3.53	0.740	2.450	0.47-12.8	0.287
Diabetes mellitus	0.318	0.0613-1.65	0.173	0.762	0.136-4.27	0.757
Platelet count	1.000	0.95-1.01	0.586	0.990	0.978-1.0	0.131
Creatinine	82.700	2.39-2860	0.015	0.994	0.67-2.69	0.991
**C reactive protein**	**1.290**	**1.090-1.53**	**0.004**	**1.170**	**1.06-1.30**	**0.003**
**Oxygenation**	7.000	1.98-24.70	0.003	4.150	0.953-18.1	0.058
**Intubation**	**14.700**	**1.7-127**	**0.015**	**11.500**	**2.31-57.3**	**0.003**
**Gastrointestinal symptoms**	**3.040**	**1.010-9.11**	**0.048**	**6.530**	**1.25-34.0**	**0.026**
Investigational agent	8.290	2.540-27.10	<0.001	2.900	0.5570-15.1	0.206

Abbreviations: OR, odds ratio; CI, confidence interval.

### 3. Gastrointestinal symptoms and pneumonia stratify the degree of liver injury

It is well known that CRP and oxygenation are associated with the severity of pneumonia. In our cohort, the main focus of COVID-19 was pneumonia. Therefore, based on these results, we investigated liver enzyme elevation at the point of admission and at peak with GS, pneumonia, and intubation cases.

In AST on admission, the degree of patients with GS, pneumonia, and intubation were significantly higher than those without these symptoms (*P =* 0.016, *P =* 0.0204, *P =* 0.00196, respectively; Mann-Whitney *U* test, Table 3). In ALT on admission, the degree of patients with pneumonia was significantly higher than those without pneumonia (*P =* 0.0212). The baseline clinical characteristics were not significantly different except the degree of inflammation (lower albumin and higher CRP) in patients with GS, pneumonia, and intubation.

**Table 3:** Baseline characteristics of patients with COVID-19 classified with or without gastrointestinal symptoms, pneumonia, and intubation.

	Gastrointestinal symptoms			Pneumonia			Intubation		
	No n = 33	yes n = 27	P value	No n = 9	yes n = 51	P value	No n = 51	yes n = 9	P value
Male gender (n, %)	19 (57.6%)	20 (74.0%)	0.587	5 (55.6%)	34 (66.7%)	0.706	32 (62.7%)	7 (77.8%)	0.473
Age (years)	56 (22–86)	57 (19–82)	0.372	50 (19–67)	57 (33–86)	0.373	53 (19–86)	62 (44–82)	0.00851
Platelet (x10^4^/μl)	20.7 (13.0–42.3)	18.5 (11.2–38.4)	0.223	21.3 (16.7–42.3)	20.1 (11.2–42.0)	0.15	20.7 (11.2–42.3)	17.4 (11.8–28.5)	0.0582
Albumin (g/dl)	4.0 (2.4–4.9)	3.3 (2.4–4.6)	0.0326	4.3 (3.7–4.9)	3.7 (2.4–4.8)	0.0013	3.9 (2.4–4.9)	3.1 (2.9–4.1)	0.042
Total bilirubin (mg/dl)	0.5 (0.2–1.2)	0.5 (0.3–2.1)	0.312	0.5 (0.3–1.2)	0.5 (0.2–2.1)	0.864	0.5 (0.2–1.2)	0.5 (0.2–2.1)	0.724
Alkaline phosphatase (IU/L)	188 (142–1060)	219 (122–590)	0.163	175 (142–225)	203 (122–1060)	0.0113	192 (122–1060)	220 (159–590)	0.0506
Creatinine (mg/dl)	0.74 (0.45–1.69)	0.82 (0.59–5.89)	0.0621	0.67 (0.59–1.04)	0.78 (0.45–5.89)	0.259	0.74 (0.45–1.10)	1.05 (0.75–5.89)	<0.001
C reactive protein (mg/dl)	1.34 (0.03–19.44)	8.83 (0.03–40.61)	<0.001	0.40 (0.03–5.66)	2.92 (0.13–40.61)	0.00351	2.32 (0.03–19.44)	16.8 (2.68–40.61)	<0.001
Aspartate transaminase (IU/L)	29 (16–141)	41 (19–430)	0.016	25 (16–48)	38 (18–430)	0.0204	32 (16–430)	69 (30–218)	0.00196
Alanine aminotransferase (IU/L)	29 (8–172)	38 (12–292)	0.212	17 (13–60)	37 (8–292)	0.0233	32 (8–292)	38 (16–127)	0.182

**Fig 1:**
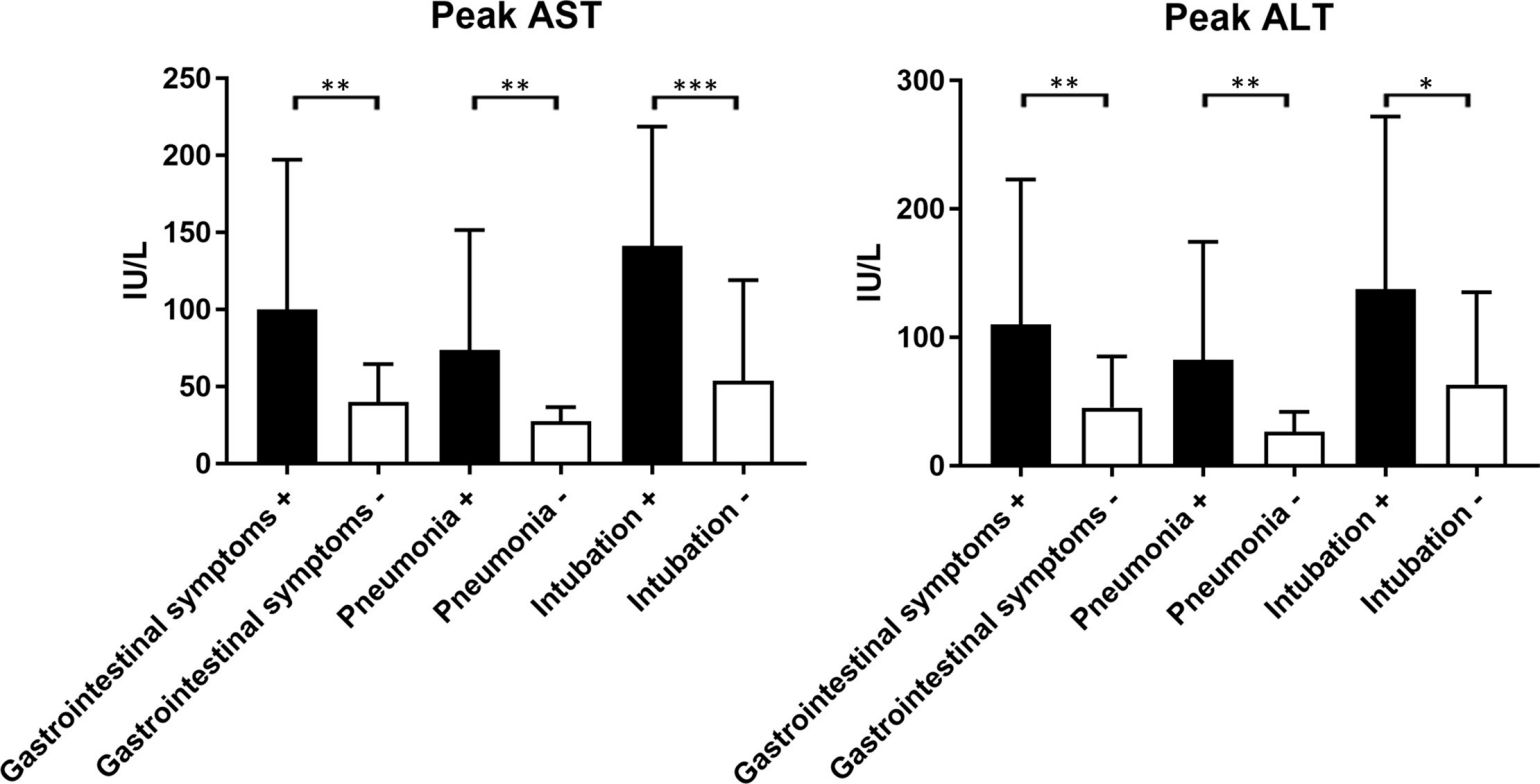
Liver enzyme at peak in patients with COVID-19 classified with or without gastrointestinal symptoms, pneumonia, and intubation; *P<0.05, **P<0.01.

In AST and ALT at peak, the degree of patients with GS, pneumonia, and intubation was significantly higher than those without (AST: *P =* 0.031, *P =* 0.0063, *P*<0.001; ALT: *P =* 0.011, *P =* 0.0025, *P =* 0.0335, respectively; Fig 1).

### 4. The changes of biochemistries of patients with gastrointestinal symptoms

**Fig 2:**
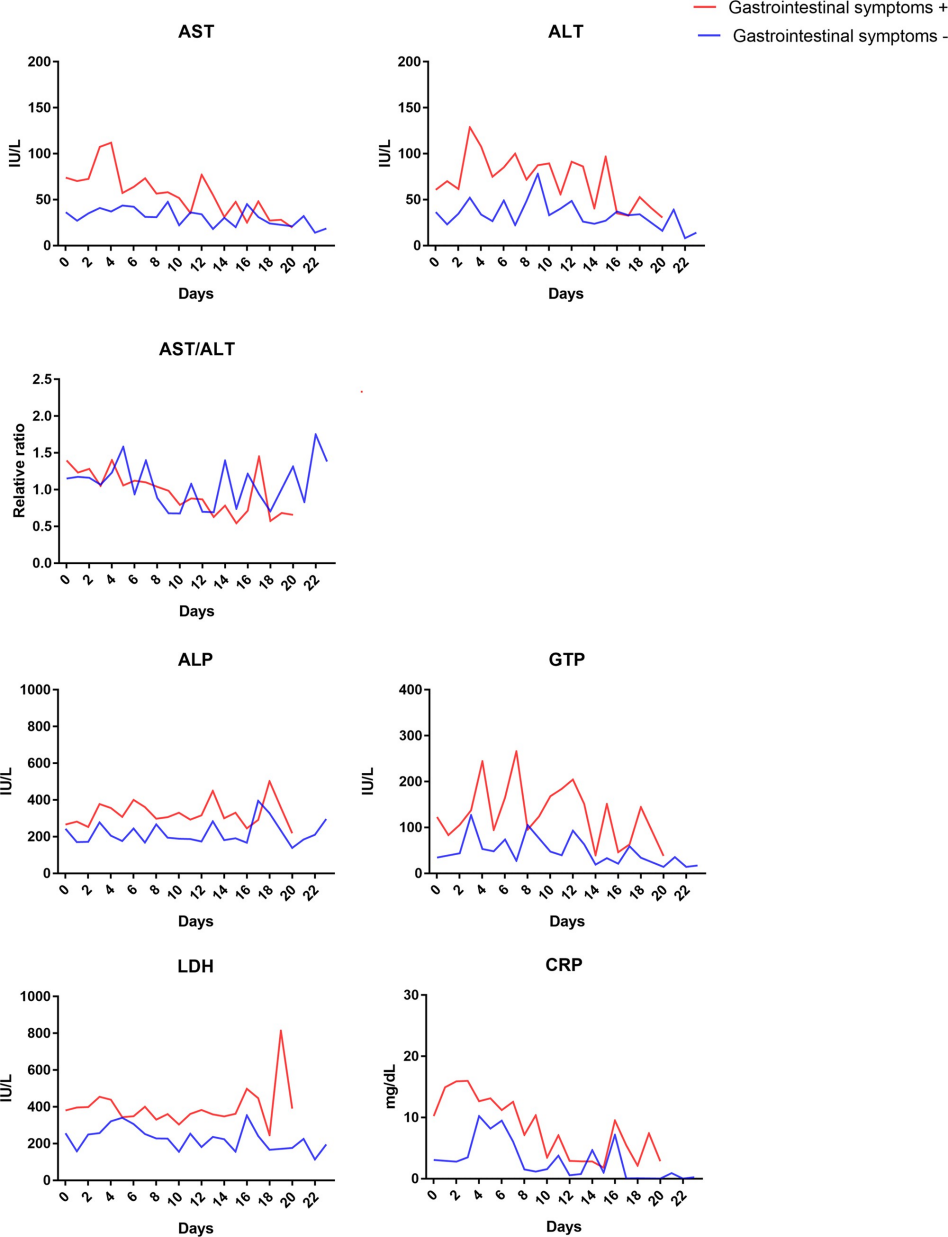
The dynamics of serum biochemistries of patients with COVID-19 with or without gastrointestinal symptoms.

Next, the dynamics of serum biochemistries of patients with COVID-19 with or without GS was investigated. Not all biochemistries were measured in each case on each day as a dataset, and the statistical comparison was impossible. However, it was presumed that not only liver enzymes (AST and ALT) but also biliary (ALP and guanosine-5'-triphosphate [GTP]), lactate dehydrogenase (LDH), and inflammatory markers (CRP) moved relatively in parallel (Fig 2).

### 5. The prediction of liver injury

**Fig 3:**
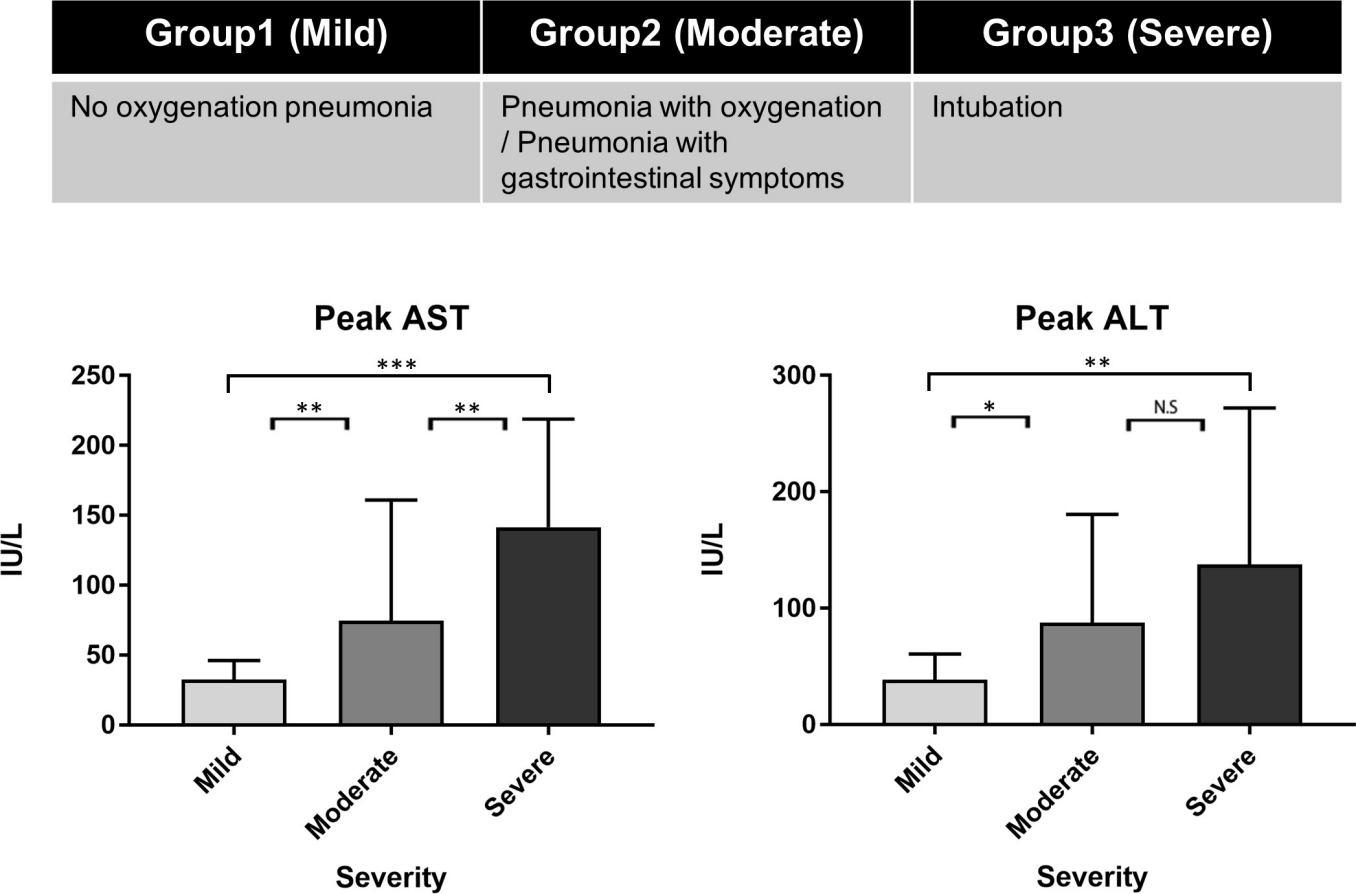
The peak of liver enzymes of patients with COVID-19 with a new severity classification for liver injury.

To predict the degree of liver injury, we classified patients with COVID-19 into three groups: group 1, no pneumonia or pneumonia without oxygenation; group 2, pneumonia with oxygenation or GS; and group 3, intubation. We classified 25 (41.7%), 26 (43.3%), and 9 (15.0%) patients into groups 1–3, respectively. The breakdown of 26 patients in group 2 was as follows: 9 patients with both oxygenation and GS, 10 patients with GS and without oxygenation, 7 patients with oxygenation and without GS. The median of AST at peak was 28 IU/L (mild), 48 IU/L (moderate), and 109 IU/L (severe) in groups 1–3, respectively, and was significantly stratified with severity (*P*<0.001, Kruskal-Wallis test). Furthermore, the median of ALT at peak was 33 IU/L, 47.5 IU/L, and 106 IU/L in groups 1–3, respectively, and was also significantly stratified with severity (*P* = 0.0114) (Fig 3).

Based on these results, liver injury was significantly stratified when divided into three groups based on GS and pneumonia status. The GS status of COVID-19 was complementary to assess liver injury.

## Discussion

This study provides the first evidence that the stratification for liver enzyme elevation based on GS and pneumonia status was possible.

Observational study [3, 9, 10], systematic review, and meta-analysis [8, 11, 12] recently reported the severity of COVID-19, GS, and liver injury. However, the definition of COVID-19 severity differs depending on reports about pneumonia [16–18]. Most of them were subgroup analysis of COVID-19 pneumonia in terms of GS and liver injury. The detailed observational study from the viewpoint of GS and liver injury was expected.

In our cohort, 27 (45.0%) patients had GS (Table 1), which was relatively more than 15% pooled prevalence of digestive symptoms in a meta-analysis [8]. In analyzing factors associated with liver enzyme elevation, we found that not only the factors about the severity of pneumonia (CRP, oxygenation, and intubation) but also GS was a significant factor associated with the elevation of liver enzyme (Tables 2 and 3 and Fig 1). These results support reports that GS was associated with liver injury in patients with COVID-19 [19, 20].

Furthermore, we made the definition about the predictive COVID-19 liver injury degrees according to the elevation of liver enzyme as follows: group 1, no pneumonia or pneumonia without oxygenation; group 2, pneumonia with oxygenation or GS; and group 3, intubation. This definition could stratify the degree of liver injury as mild, moderate, and severe in groups 1–3, respectively (Fig 3).

However, attention should be paid to describe the expression “liver injury.” It is controversial whether liver enzyme elevation is related to a specific liver disorder caused by COVID-19. Some reports revealed that the angiotensin-converting enzyme 2, as SARS-CoV-2 entry receptor [2], was expressed in hepatocytes, cholangiocytes, and gastrointestinal tract [21, 22]. There were also some reports about fecal-oral transmission of SARS-CoV-2 [23–25]. Thus, the virus may infect the liver; however, there is no evidence that its life cycle was reproduced in the liver because of insufficient study reports. In this study, some investigational agents such as ciclesonide, favipiravir, and hydroxychloroquine were used. The EASL-ESCMID review summarized the drugs for COVID-19 and liver injury [26, 27]. Hydroxychloroquine has not been associated with liver injury. Ciclesonide cause liver enzyme elevation less than 1%. Favipiravir could happen elevation of AST and ALT. From the point of view, favipiravir may cause drug induced liver injury. However, patients who received investigational agents have already had liver injury and relatively severe COVID-19 on admission as shown in S1 Table. The liver injury with CTCAE Grade >2 at peak was not significantly associated with investigational agent administration (Table 2). Regarding liver injury, distinguishing between cytokine storms associated with viral infection and drug-induced liver injury is difficult [28, 29], and the COVID-19-specific pathological findings are still unclear. Further reports about liver biopsy and autopsy are expected.

We also investigated the dynamics of biochemistry (Fig 2). It was suggested that liver enzyme moved relatively parallel to other markers, such as biliary enzymes (ALP, GTP), LDH, and inflammatory markers (CRP). There were valuable data because there were few reports about the dynamics of biochemistry [30].

However, this study had several limitations. First, this was a retrospective study in which the exclusion of unidentified biases was impossible. Second, we were unable to analyze the dynamics of biochemistry statistically. If possible, the same biochemistry on the same schedule should be collected. Lack of data and serum samples precluded the performance of such analyses here. Third, although the study indicated the usefulness of GS for predictive liver enzyme elevation as a clinical setting report, this result was mostly in Japanese populations, which may not be able to adapt to racial differences. In terms of clinical implementation, it would be better if we were able to reveal the predictive performance of GS and a new severity classification for the need of a ventilator and the prognosis of COVID-19. The observation period and number of patients may be insufficient to make a solid comparison and to reveal such evidence. A follow-up study using a larger cohort with this classification system is needed in the future.

In conclusion, GS was useful for stratifying the elevation of liver enzyme in patients with COVID-19. Furthermore, the novel classification system, which combined GS, oxygenation, and intubation status, was valuable to provide a more detailed stratification. The application of GS assessment may lead to the early detection of severe course, helping to reveal liver injury mechanism caused by COVID-19.

## Supporting information

S1 Table Characteristics of patients with COVID-19 classified with or without investigational agent. (DOCX)
