# Supplementary material for: Liver injury with COVID-19 based on gastrointestinal symptoms and pneumonia severity
Source: PLoS One. 2020 Nov 4;15(11):e0241663. doi: 10.1371/journal.pone.0241663 (PMC7641400; doi:10.1371/journal.pone.0241663)
Supplement: S1 Table — (DOCX) [file pone.0241663.s001.docx]

**S1 Table.** Characteristics of patients with COVID-19 classified with or without investigational agent

|  | Investigational agent | | |
| --- | --- | --- | --- |
|  | No n=23 | yes n=37 | P value |
| Male gender (n, %) | 15 (65.2%) | 24 (64.9%) | 0.999 |
| Age (years) | 52 (19-86) | 58 (33-82) | 0.146 |
| Platelet (x10^4^/µl) | 20.0 (13.0-42.3) | 20.4 (11.2-35.5) | 0.939 |
| Albumin (g/dl) | **4.0 (3.1-4.9)** | **3.6 (2.4-4.8)** | **0.0167** |
| Total bilirubin (mg/dl) | **0.4 (0.2-1.2)** | **0.6 (0.3-2.1)** | **0.0295** |
| Alkaline phosphatase (IU/L) | **183 (122-253)** | **220 (142-1060)** | **0.000613** |
| Creatinine (mg/dl) | 0.70 (0.49-1.10) | 0.79 (0.45-5.89) | 0.109 |
| C reactive protein (mg/dl) | **1.94 (0.03-14.7)** | **4.59 (0.13-40.61)** | **0.0109** |
| Aspartate transaminase (IU/L) | **28 (16-75)** | **40 (18-430)** | **0.00241** |
| Alanine aminotransferase (IU/L) | **22 (8-90)** | **38 (13-292)** | **0.0106** |
